# eQTL-Detect: nextflow-based pipeline for eQTL detection in modular format with sharable and parallelizable scripts

**DOI:** 10.1093/nargab/lqae122

**Published:** 2024-09-24

**Authors:** Praveen Krishna Chitneedi, Frieder Hadlich, Gabriel C M Moreira, Jose Espinosa-Carrasco, Changxi Li, Graham Plastow, Daniel Fischer, Carole Charlier, Dominique Rocha, Amanda J Chamberlain, Christa Kuehn

**Affiliations:** Research Institute for Farm Animal Biology (FBN), Wilhelm-Stahl-Allee 2, 18196 Dummerstorf, Germany; Research Institute for Farm Animal Biology (FBN), Wilhelm-Stahl-Allee 2, 18196 Dummerstorf, Germany; Unit of Animal Genomics, GIGA Institute, University of Liège, 4000 Liège, Belgium; Centre for Genomic Regulation (CRG), The Barcelona Institute of Science and Technology, Dr. Aiguader 88, Barcelona 08003, Spain; Department of Agricultural, Food and Nutritional Science, University of Alberta, Edmonton T6G 2P5, Canada; Lacombe Research and Development Centre, Agriculture and Agri-Food Canada, T4L 1W1 Lacombe, Canada; Department of Agricultural, Food and Nutritional Science, University of Alberta, Edmonton T6G 2P5, Canada; Natural Resources Institute Finland (Luke), Green Technology, Animal and Plant Genomics and Breeding, FI-31600 Jokioinen, Finland; Unit of Animal Genomics, GIGA Institute, University of Liège, 4000 Liège, Belgium; Université Paris-Saclay, INRAE, AgroParisTech, GABI, 78350, Jouy-en-Josas, France; Agriculture Victoria Research, AgriBio, Centre for AgriBiosciences, Bundoora, VIC 3083, Australia; School of Applied Systems Biology, La Trobe University, Bundoora, VIC 3083, Australia; Research Institute for Farm Animal Biology (FBN), Wilhelm-Stahl-Allee 2, 18196 Dummerstorf, Germany; Faculty of Agricultural and Environmental Science, University Rostock, Justus-von-Liebig-Weg 6, 18059 Rostock, Germany; Friedrich-Loeffler-Institut (FLI), Federal Research Institute for Animal Health, 17493 Greifswald, Insel Riems, Germany

## Abstract

Bioinformatic pipelines are becoming increasingly complex with the ever-accumulating amount of Next-generation sequencing (NGS) data. Their orchestration is difficult with a simple Bash script, but bioinformatics workflow managers such as Nextflow provide a framework to overcome respective problems. This study used Nextflow to develop a bioinformatic pipeline for detecting expression quantitative trait loci (eQTL) using a DSL2 Nextflow modular syntax, to enable sharing the huge demand for computing power as well as data access limitation across different partners often associated with eQTL studies. Based on the results from a test run with pilot data by measuring the required runtime and computational resources, the new pipeline should be suitable for eQTL studies in large scale analyses.

## Graphical Abstract

**Graphical Abstract:**
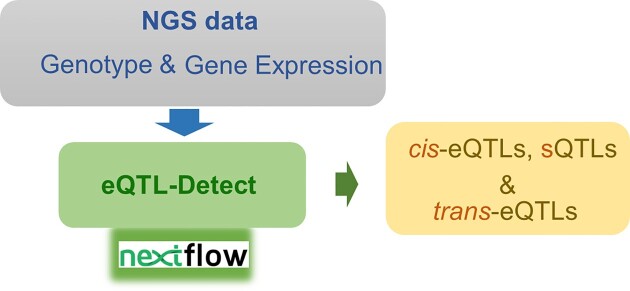

## Introduction

A computing pipeline or workflow can be defined as a series of tasks carried out in a sequential or parallel fashion with single or multiple software tools channelling output from one process as input into next processes. To this end it is set up to generate targeted output after taking input data such as raw sequence reads. In the context of analysing genomic data, these computing workflows are called bioinformatic pipelines and are especially designed to analyse high-throughput omics data like genomics, transcriptomics, proteomics, phenomics or metabolomics. As a result, they provide a comprehensive understanding of the processed input data in the form of reports, plots and statistical inferences. Initially, bioinformatic pipelines were simple with fewer tasks and dealt with smaller amounts of raw data than what we see today. Also, often they were written using simple adhoc Bash or Perl scripts. But new approaches are required now due to the ever increasing amount of NGS data generated from assays exploring (genome-wide) replication, transcription, translation, methylation, etc. combined with new software tools and techniques that require multiple pre- and post-processing steps (1). Moreover, the processing time and the required computational resources for data analysis have substantially increased, which generates an additional layer of computational complexity. Although it is possible to write correspondingly complex scripts using Linux Bash, processing such large amount of data with a series of Bash scripts is extremely inconvenient, as it requires constant tracking of intermediate log files and monitoring of the running processes. Also, it is difficult to (re)start such a pipeline from the point, where it stopped, if there are any interruptions. As a consequence it is often necessary to re-run the entire pipeline from the beginning to avoid possible conflicts with intermediate files. A further challenge complex scripts frequently encounter is the need to include code snippets from other programming languages like Python and R to run some specific tasks within a pipeline. This requires calling the code snippets within the Bash scripts. Furthermore, each time a script is re-run with a different input on a different computational system, care should be taken regarding the software used, the versions of software packages installed, and the location where the data is stored. All these challenges make it very difficult to reproduce the results of a computational analysis (2). Bioinformatic pipelines developed with workflow manager technologies like Snakemake (3), Common Workflow Language (4), Nextflow (5), etc. overcome most of these issues with relatively simple coding, and the pipelines resulting from them are portable, scalable and provide reproducible results (6). Such workflow managers allow the user to design complex pipelines with minimal coding and seamless movement of data from one step to the next. Furthermore, they keep track of all the running tasks and can be resumed from the point they stopped in case of any unforeseen interruption. Most of the workflow managers also allow deployment of pipelines in cluster and cloud computational environments, which enable different tasks to be run in parallel and in a distributed format. Finally, workflow managers support container technologies like Docker (https://www.docker.com/) and Singularity (https://docs.sylabs.io/guides/3.5/user-guide/introduction.html) for installing all the software tools required for a pipeline. Distributing software versions and dependencies in containers guarantees the reproducibility and portability of pipeline results regardless of the underlying computational environment.

In the current study, we developed a bioinformatic pipeline for eQTL detection with the primary goal of identifying eQTLs in a large international network project (EU Horizon 2020-funded BovReg, https://bovreg.eu/) with contributions from many laboratories and partners. eQTL studies identify genome-wide genetic variants that could regulate gene expression (7,8). The availability of huge amounts of genetic variants and expression data provides the opportunity to conduct high-resolution eQTL studies with millions of genetic variants and thousands of genes in hundreds to thousands of samples across multiple tissues, which gives adequate statistical power for statistically significant eQTL estimates. In international projects like BovReg, multiple partners come together with high-quality input data from large datasets. However, conducting analyses with such a large amount of data is a huge computational burden. Moreover, a complex bioinformatic pipeline like eQTL detection contains several pre-processing steps of raw input data, which further adds high volumes of intermediate data. Thus, in collaborative studies it would be more convenient for the contributing teams to pre-process the data, which would distribute a substantial part of the computational burden among partners. Furthermore, sometimes due to some technical issues or legal constraints regarding full data availability, the initial raw data generated are not sharable between partners of a project. To overcome such issues and considering different usable scenarios to run the pipeline, we provided two options, either to run the entire pipeline with a single command by using a single nextflow script for single users or by distributing the tasks of the pipeline into three independent nextflow modules which facilitates the distribution of the pipeline tasks among multiple users. This modular design facilitates the confidential distribution of the computational workload among partners to pre-process the raw input data. We considered Nextflow (5) as a base to develop the pipeline as it offers the flexibility to define individual tasks as separate files called ‘Modules’ and enables a group of modules to be combined to form sub-workflows. To estimate the required amount of computational resources and runtime for actual analyses with BovReg data, a pilot run was carried out with whole genome sequence (WGS) genotype data and liver RNA-seq output for 88 individuals. A subset of the dataset from the pilot run is available for testing along with the scripts in the BovReg GitHub page (https://github.com/BovReg/BovReg_eQTL) for potential users of the pipeline.

## Materials and methods

### Pipeline and implementation

The eQTL-Detect bioinformatic pipeline was designed for detecting eQTL using the new DSL2 (domain specific language 2) Nextflow modular syntax and executed using Nextflow (5). All the software with versions used for this pipeline are indicated in Table 1. To make our pipeline portable to other computational environments, all the software required to run our pipeline are made available in Docker Hub (https://hub.docker.com/u/praveenchitneedi). We provided different nextflow config files in the folder ‘conf’ (https://github.com/BovReg/BovReg_eQTL/tree/main/conf) to run the pipeline either on a local server or on a high performance computing clusters like Slurm (https://slurm.schedmd.com/documentation.html), PBS (https://www.openpbs.org/) or SGE (http://star.mit.edu/cluster/docs/0.93.3/guides/sge.html). Moreover, in addition to Docker, this pipeline also supports Singularity and Podman (https://podman.io/) container technologies to install all the software required to run the pipeline. To improve the ease of use, all the required parameters to run this pipeline with default values and also the paths for the input and output data are declared in a single file ‘nextflow.config’ (https://github.com/BovReg/BovReg_eQTL/blob/main/nextflow.config). This pipeline can be replicated in any computational environment by exporting the required configuration files and scripts, provided Nextflow and any of the above mentioned container technologies are installed. During execution of each process in these scripts, Nextflow pulls the corresponding software with specified version from Docker hub. As a result, it runs the pipeline with the exact tool versions so that users just have to take care of declaring the correct paths of input data and the location, where the output should be stored. Nextflow stores all log files and output data in a directory named as ‘work’. Generally based on the number of processed samples the size of this ‘work’ directory increases substantially during the runtime of the script. Thus, care should be taken by allocating enough storage space before running the pipeline. Since this directory is used to resume or re-run the pipeline with alternative parameters, once the analysis is done and the results are stored in a separate folder, this directory can be deleted, if no demand for resuming or re-running the pipeline with alternative parameters is intended.

**Table 1.** This table shows versions of the different software used in the eQTL-Detect pipeline and also web links for some software which explains about specific software parameters used in this pipeline

Software/parameter	Version/webpage
Nextflow	22.10.6.5843
Docker	20.10.8, build 3967b7d
Singularity	3.11.4
Podman	3.4.4
fastqc	0.11.9
STAR	2.7.0d
trimmomatic	0.36
Samtools	1.9–4
StringTie	1.3.3
RegTools	0.6.0
LeafCutter	0.2.9
VCFtools	v0.1.12b
csvtk	0.21.0
csvtk–merge	https://bioinf.shenwei.me/csvtk/usage/
SNPRelate	1.34.1
QTLtools	1.3
QTLtools *cis* nominal	https://qtltools.github.io/qtltools/pages/mode_cis_nominal.html
QTLtools *cis* permutation	https://qtltools.github.io/qtltools/pages/mode_cis_permutation.html
QTLtools *cis* conditional pass	https://qtltools.github.io/qtltools/pages/mode_cis_conditional.html
QTLtools trans full pass	https://qtltools.github.io/qtltools/pages/mode_trans_full.html

**Figure 1.**
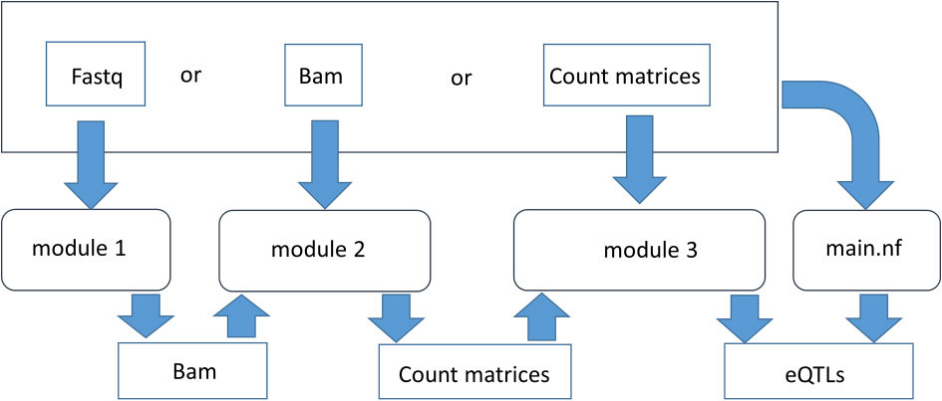
The flow of input expression data in different formats (fastq, bam or count matrices) after execution from different modules and from standalone script main.nf. Modules can take the expression data generated from previous modules or generated externally. The standalone script accepts any of these three formats as input and runs the entire analyses until eQTL detection.

The input data for the pipeline consists of (i) files from RNA-seq experiments either as raw sequence in fastq file format, which can be either single-end or paired-end reads, or aligned and sorted files in bam format or normalized expression count matrices (gene level, transcript level and splicing sites) across different samples in TSV (Tab-separated values) format, (ii) WGS data as genotypes in Variant Call Format (VCF) (https://samtools.github.io/hts-specs/VCFv4.2.pdf, last accessed in July 2024) and (iii) reference genome information of the species of interest for RNA-seq read alignment (genome assembly and corresponding transcriptome annotation files) to establish the phenotype count matrices. Users should declare the paths of all the input samples (expression data and genotype data) as sample sheets in ‘.tsv’ format, users can access the example sample sheet files from the folder ‘Demodata’ (https://github.com/BovReg/BovReg_eQTL/tree/main/Demodata). By default this pipeline takes fastq files with paired-end reads as input RNAseq samples, users can declare an alternative expression input files (single-end fastq files or aligned and sorted Bam files or expression count matrices) in the nextflow.config. The entire pipeline can be executed using a single standalone script main.nf by using the command *nextflow run main.nf -c conf/env_local.config -profile docker* or by using three separate modules (module_1_eQTLDetect.nf, module_2_eQTLDetect.nf and module_3_eQTLDetect.nf) using the command *nextflow run module_(1 or 2 or 3)_eQTLDetect.nf -c conf/env_local.config -profile docker* (Figure 1). The module 1 performs reference genome indexing, quality check of input RNAseq reads, aligning the reads and sorting them in bam format. If aligned and sorted bam files are available, users can skip the execution of module 1 and can directly use module 2 to generate normalized count matrices, for the available genotyped samples. This module extracts the genotyped sample for the available corresponding RNAseq samples and also generates expression counts at gene level, transcript level and for splicing sites. The final module 3 detects eQTLs with the expression count matrices and corresponding genotypes as input for the available samples, if these files are available users can just use this module. This module extracts the genotyped samples having corresponding RNAseq samples, performs principal component analysis (PCA) both for genotype and expression data and finally detects *cis-* and *trans*-eQTLs.

**Figure 2.**
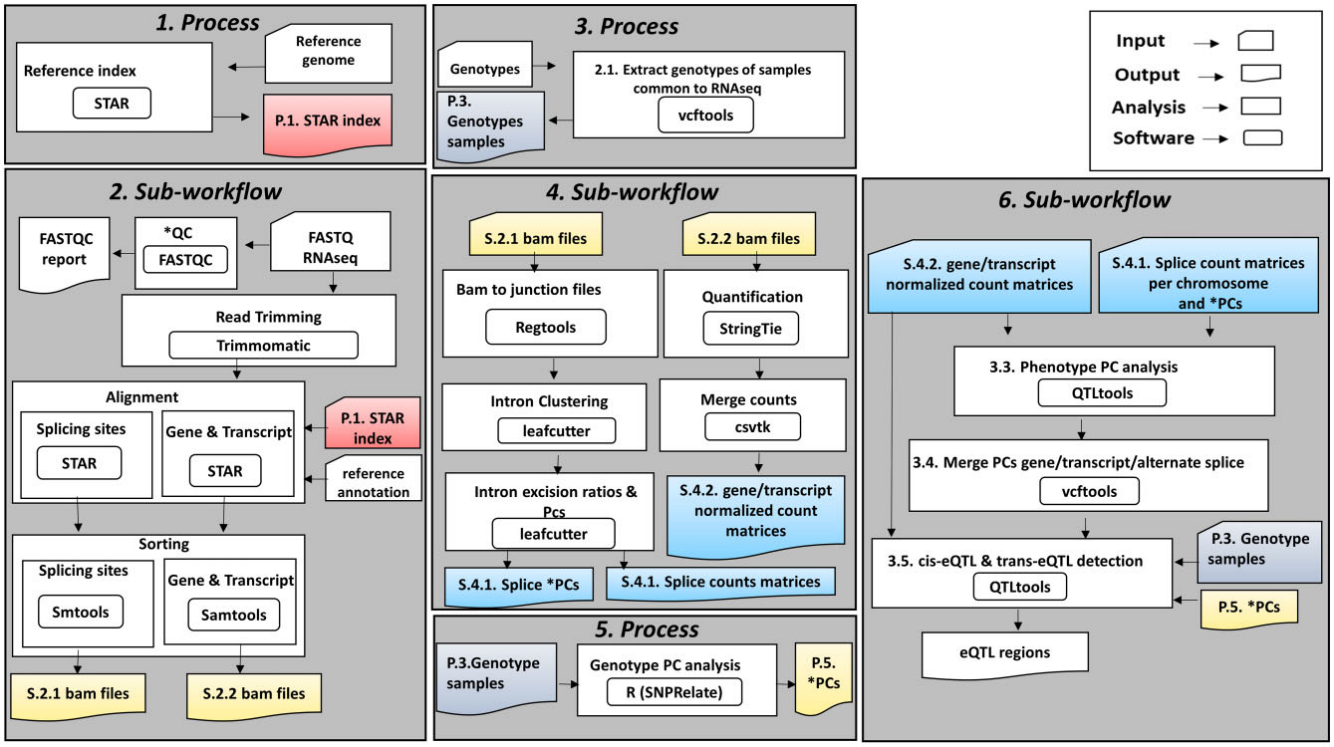
eQTL-Detect bioinformatics pipeline: each grey block represents an independent Nextflow process or sub-workflow. Some input/output boxes are coloured to represent the flow of data between processes and sub-workflows, example: ‘P.1. STAR index’ coloured in red to represent the reference genome STAR index output from 1. Process is used as input in 2. Sub-workflow. The shape of individual entities in grey blocks refers to input, output, analysis or software as shown at the top right of the figure. *QC, quality control; PCs, principal components.

Each individual analysis in the nextflow pipeline is defined as an independent entity called ‘Process’, and they communicate with other processes via input and output components called ‘Channel’. The input data declared in the nextflow.config file are also feed to different processes via channels. To increase the usability, we adopted the concept of nextflow dsl2 subworkflow design for some tasks like expression data processing and eQTL detection. (https://github.com/BovReg/BovReg_eQTL/tree/main/subworkflows). For processing the expression data two separate sub-workflows are designed, one for paired-end reads and other single-end reads. A separate sub-workflow is designed for expression data quantification and creating count matrices. Finally for the detection of *cis-*eQTL and *trans*-eQTL takes place in two separate sub-workflows. The required processes and sub-workflows are invoked while executing the main.nf and the three modular scripts based on the parameters declared in the nextflow.config file. All the important results from each process are stored in separate pre-defined folders in a user defined output path. The details of all the processes and sub-workflows of the pipeline was shown in Figure 2 and described below.

1. *Process: genome indexing*. This process performs the indexing of the reference genome provided by the user. As a prerequisite to perform read alignments using STAR (9), the indexing of a reference genome using the STAR command ‘–runMode genomeGenerate’ is required. The indexed reference genome acts as reference for subsequent read alignment of all RNA-seq samples.
2. *Sub-workflows: processing paired-end and single-end reads*. The two sub-workflow defined for paired-end and single-end reads performs the pre-processing of RNA-seq. Initially, a quality check of the input reads in each RNA-seq sample is performed followed by trimming of poor quality and adapter sequences. Then aligning the trimmed reads against the reference genome, followed by sorting and indexing that aligned samples.
  The input read quality testing is performed by FASTQC (10). Using the software trimmomatic (11), we implemented the trimming of paired-end and single-end reads as separate processes in each sub-workflow. Trimmed reads of each sample are aligned against the reference genome using STAR (9) in combination with the corresponding transcriptome annotation file in General Transfer Format (GTF) format, to perform an annotation-guided alignment. We used two different sets of parameters for aligning RNA-seq reads using STAR corresponding to the different eQTL phenotypes: one set serves to identify gene and transcript regions and other for identifying splicing sites. The input data for alignment in each sub-workflow are handled differently for paired-end or single-end reads. In case of paired-end reads, the input data include paired reads and unpaired reads without mate after trimming, and these two sets are aligned separately to the reference genome. Later, the resulting aligned reads are merged by the software Samtools (12). For the genes and transcript regions the reads are sorted by coordinates during alignment and for detecting splice junctions during alignment, the reads are kept unsorted and the parameters ‘–twopassMode Basic’ and ‘–outSAMstrandField intronMotif ’ are used to determine, if the read origin is from intron or from splice junction. The unsorted reads are indexed and sorted using Samtools.
3. *Process: extracting genotype samples have corresponding expression samples*. This process collate the set of genotype files comprising samples with expression data available. It takes the genotype data per each chromosome individually in VCF format for the samples available for the eQTL analysis. The genotype data are quality-filtered at individual level and SNP level using plink (13) by filtering out the sample having more than 1% missing genotypes (–mind 0.01) and filtering out the SNP genotypes with the plink filters *–maf 0.005 –geno 0.5 and –hwe 1e-6*. The list of respective samples of the input VCF file is intersected with the list of samples with RNA-seq data to generate a text file having the corresponding RNA-seq sample names. With this text file, the genotypes from the input VCF file are filtered for all samples having both RNA-seq and genotype data using custom Linux commands and VCFtools (14). To enable eQTL analysis across data sets from different origins, the user has to take care that the assignment of the reference and alternate alleles for each genotype in the VCF file is the same as defined in the reference genome assembly used for aligning the RNA-seq reads.
4. *Sub-workflow: generation and merging expression count matrices*. This sub-workflow has a set of processes for quantifying the expression counts per each sample at gene level, transcript level and splicing events (based on intron excisions (15)) according to information found in the annotated reference genome and merging all the expression counts per each sample into count matrices The StringTie (16) assembler quantifies and normalizes the read counts at gene and transcript level, accounting for strandedness of the RNA-seq library with option ‘–rf’ for the first stranded libraries, ‘–fr’ for the second stranded libraries and no parameter for unstranded libraries. For quantifying the splicing sites, exon-exon junctions are extracted from the indexed bam files using RegTools (17) ‘junctions extract’, and the appropriate strandedness of the RNA-seq library is taken care of with the regtools option ‘-s 1’ for the first stranded libraries, ‘-s 2’ for the second stranded libraries and ‘-s 0’ for unstranded library. After the sample wise quantification of expressions, the phenotype input files are generated at gene and transcript level by generating the count matrices from the StringTie quantification output files for individual samples. The normalized TPM values from the samples with genotype data are retained. The counts across all gene/transcripts for the filtered list of samples are then merged into a matrix format with samples as columns and gene/transcripts expression counts as rows using csvtk –merge (Table 1) and are stored in Tab-Separated Values (TSV) format. From this table, genes/transcripts are filtered out, if they do not have TPM values greater than one in at least 10 percent of samples. These tables are provided as input files for the detection of gene eQTL (geQTL) and transcript eQTL (teQTL).The last process in this sub-workflow generates the count matrices for splicing events across different samples for each chromosome using two custom Python scripts from the LeafCutter package (15). The first python script (leafcutter_cluster_regtools.py) performs the clustering of introns found in the exon-exon splice junction files generated in script 1. The second python script (prepare_phenotype_table.py) calculates intron excision ratios filtering out introns present in <40% of individuals or with no variation and finally, it delivers files with the intron excision ratios for each chromosome separately across all samples along with the user defined principal components (PCs). These intron excision ratios generated across samples form the splicing phenotypes and the PC estimates are covariates for the splice eQTL (sQTL) detection.
5. *Process: genotype PCA*. The genotype covariates address the population stratification present due to systematic ancestry differences in the data set and are included by performing a PCA with genotype input using the R package SNPRelate (18). This process takes the genotype data in VCF format and the number of PCs declared as parameter in nextflow.config file to perform PCA. The output PCA file is supplied to sub-workflow using nextflow channel as input for detecting *cis-* and *trans*-eQTLs.
6. *Sub-workflow: cis- and trans-eQTL detection*. Two separate sub-workflows are developed for performing *cis* and *trans* eQTL mapping, but both perform similar processes and both *cis-* and *trans*-eQTL mapping is by using QTLtools (19). QTLtools expects three input files for mapping: (i) genotype data in VCF format, (ii) phenotype data in bed format and (iii) the genotype and phenotype PCs as covariates. Both the sub-workflows carry out eQTL mapping per chromosome by taking the WGS genotype data and TPM normalized read counts from a particular chromosome. In case of *cis*-eQTL mapping, we selected a default 1Mb flanking window for genetic variants positioned on either side of the phenotype. In contrast, for *trans*-eQTL the TPM normalized read counts from each gene/transcript expression or splicing events are correlated with the genotype variants from each chromosome except a 5 Mb flanking window on either side of the target gene/transcript/splicing event. The input data should contain only the samples having both genotypes and the corresponding phenotype data along with the covariate text file of these samples. To address outlier samples and the batch effects in the RNA-seq data, we used QTLtools PCA to generate PCs to be used as covariates for gene and transcript level phenotypes. Although the probabilistic estimation of expression residuals (PEER) factor method is the de facto method to determine hidden variables in many eQTL studies (20–23), PCA is easier to implement and interpret. Moreover, in a recent study by Zhou *et al.* (24), it was demonstrated that PCA produces results faster, which were identical or better than PEER estimates. In the case of sQTL, the leafcutter PC estimates from script 3 are considered as phenotype covariates and script 4 directly takes those estimates as input.

The *cis*-eQTL mapping has three main steps for determining statistical significance of a potential association: nominal, permutation and conditional analyses, all these steps take the genotype, phenotype and covariates as input. The QTLtools *cis* nominal analysis takes the parameters ‘–nominal’ and ‘–normal’ (Table 1). The default nominal threshold declared in this pipeline is ‘–nominal 0.01″, which filters the phenotype-variant pairs with a nominal *P*-value below 0.01, and the parameter ‘–normal‘ enforces the phenotypes to match a normal distribution N(0,1). The permutation run gives the adjusted *P*-value between the *cis-*associated phenotypes and top variants. The QTLtools *cis* permutation run is declared with the parameter ‘–permute’ (Table 1). By default, this command performs 1000 permutations. The *cis*-eQTL conditional analysis identifies the number of independent QTL signals for a given phenotype and determines the best candidate variant per signal. The QTLtools *cis* conditional pass runs in two steps (Table 1), at first a nominal *P*-value threshold is estimated for each molecular phenotype from the permutation hits. Then in the next step, multiple forward stepwise and backward pass linear regressions are performed to identify independent signals per phenotype and to allocate the non-independent signals to those. The QTLtools *cis* conditional analysis run is invoked by using the parameter ‘–mapping’.

Similar to *cis*-eQTL detection, the *trans*-eQTL also has three steps to provide levels for determining statistical significance of a potential association: nominal, permutation and false discovery rate (FDR) estimation for each nominal hit. The nominal and permutation steps take the genotype, phenotype and covariates as input. We implemented the QTLtools trans full pass approach (Table 1) in this pipeline. The QTLtools trans nominal run includes the parameters ‘–nominal‘, ‘–normal’ and ‘–threshold’ along with the input files (genotype, phenotype and covariates). The default threshold option ‘–threshold 1e-5’ prints the hits with P-value less than 1e-5. The default option for this pipeline are ‘–permute 100’ to run 100 permutations and prints all the hits. As the trans-eQTL analysis is substantially more time-consuming than *cis*-eQTL analyses, the default permutations are restricted to 100, but users can change this number to perform more or less permutations. Finally, the FDR estimation was conducted using the R script provided by QTLtools, which takes the nominal and permutation hits from the previous two steps as input.

The default options in all scripts are declared in nextflow.config file for both, *cis* and *trans*-eQTL analyses, which can be modified based on user requirements.

## Results and discussion

### Application

We tested the functionality of this eQTL pipeline with a pilot dataset from 88 bovine liver RNA-seq samples and corresponding WGS genotype data. Testing with this small dataset was done to estimate how much runtime is required for a set of samples and the potential pitfalls to consider when running this workflow with much larger number of samples. We used only paired-end RNA-seq reads for this pilot run, but this pipeline also accepts single-read RNA-samples as input data.

For this test run using the pilot data, we built the STAR index for the latest bovine genome ARS-UCD1.2 with the Y chromosome assembly (https://www.ncbi.nlm.nih.gov/datasets/genome/GCF_000003205.7/, last accessed in July 2024) used for Run 7 of the 1000 bulls project (25). We used a custom reference transcriptome annotation file (26) in GTF format created for the BovReg project to perform an annotation guided alignment of reads and also for quantification at gene and transcript level. This annotation provided a substantially higher number of genes (47 914), transcripts (290 707) and exons (3 093 832) compared to the Ensembl annotation (https://ftp.ensembl.org/pub/release-109/gtf/bos_taurus/, last accessed in July 2024), which has 27 607 genes, 43 984 transcripts and 433 820 exons. This new annotation facilitated identification of eQTL signals for some novel genes, but the higher number of transcripts in the BovReg annotation compared to the *Bos taurus* annotation in Ensembl resulted in an extended run time for alignment. The RNA-seq samples include paired-end (2 × 100 bp) library sequences from liver samples (PRJEB34570, PRJEB33849) performed on an Illumina HiSeq 2500 system (27,28). The average number of input reads across the 88 RNA samples was 56 million read pairs varying from a minimum of 43 to 74 million read pairs. The input genotypes for this test run were imputed from medium density (50K SNPs) to high density (777K SNPs) and then to whole genome sequence (WGS) level using a step-wise imputation strategy (29) by taking the 1000 bulls genome project population Run 7 (25) as reference. The reference and alternate alleles of the imputed genotypes were confirmed to be in agreement with the reference assembly used for RNAseq alignment. The WGS genotype data set included 19 590 389 bi-allelic variants across 29 autosomal chromosomes.

We performed the *cis-*eQTL mapping with the top ten genotype and phenotype PCs as covariates and used the default options for the nominal and permutation analyses. For the *trans*-eQTL mapping, we considered the top 10 genotype and phenotype PCs as covariates and set default options for the nominal and permutation analyses. Executing a workflow with large samples requires a rough estimation of runtime on a given computer infrastructure, as it takes from a few days to months to finish the analyses depending on the input sample size. Thus, with the current demo of our eQTL workflow, we also checked the average runtime for 88 RNA-samples by the different processes on our local server.

**Figure 3.**
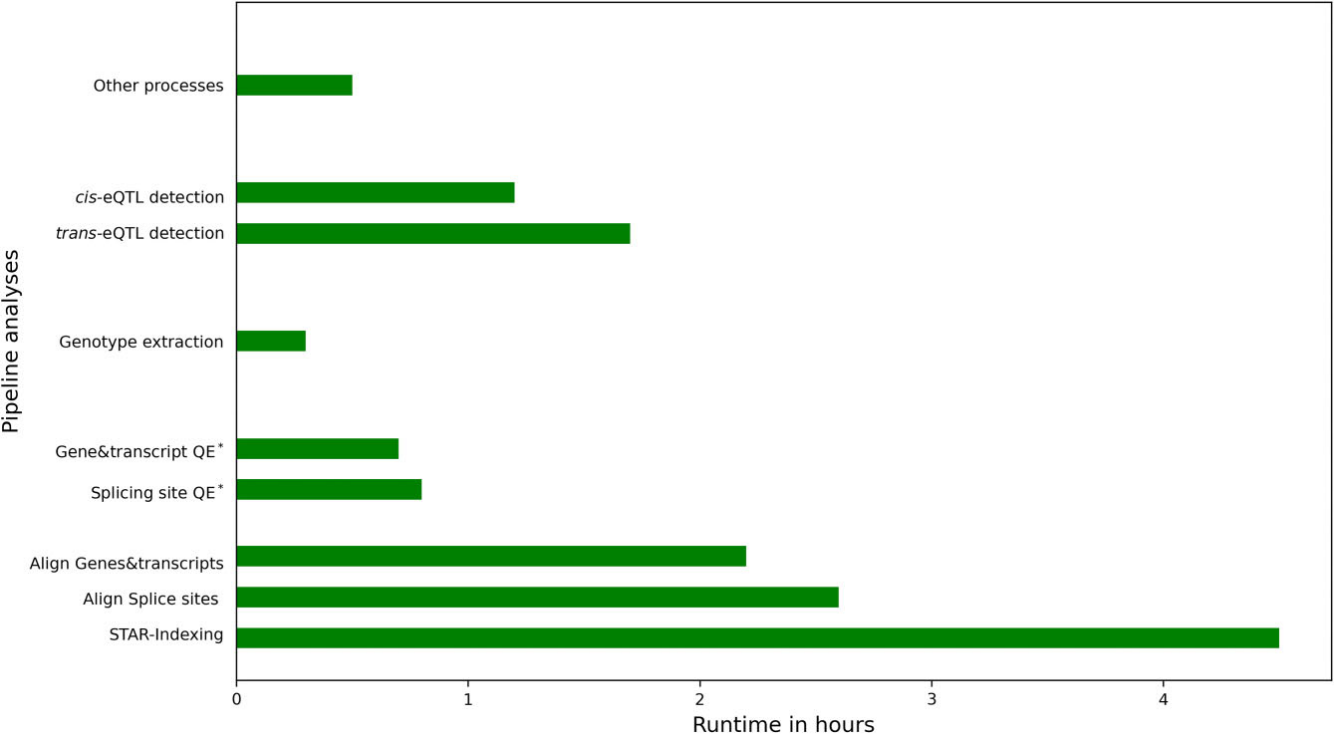
Runtime estimates of different analyses of the eQTL-Detect workflow on a server Intel(R) Xeon(R) @ 2.10 GHz server with 144 CPUs, 18 cores and 1.8 TB RAM memory: STAR indexing of bovine genome ARS-UCD1.2. Alignment and quantification for a single RNA-seq sample (2 × 100 bp) with sample size of 5.4 Gb containing 47.5 million input reads (average total read length 195). Genotype extraction of bovine chromosome 2 with 979 866 bi-allelic SNPs from 88 samples. eQTL detection and other steps in the workflow with input data for bovine chromosome 2 and expression counts from 88 RNA-seq samples. *QE, quantification of expression counts.

This test analysis was run on an Intel(R) Xeon(R) @ 2.10GHz server with 144 CPUs, 18 cores and 1.8 TB RAM memory. In this pilot run, we tested the runtime using the single command *main.nf* and also with three modular scripts, no significant change in relation to run times for running the entire pipeline using both these approaches was detected. The alignment was the most time consuming process, and aligning all 88 samples took 118 h. The quantification and merging the normalized expression counts took 9 min 15 s. It took 1 min 10 s and 1 min 35 s, respectively for extracting the genotype and generating PCs for genotype data. Finally the phenotype PCA, QTL mapping across different levels (gene, transcript and splicing events) completed in 6 h 12 min for *cis*-eQTL detection (both nominal and permutation analyses) and 7 h 21 min for *trans*-eQTL detection (both with nominal and permutation analyses). To avoid any input output biases with multiple samples and chromosomes with varied lengths, in Figure 3, we provided the runtime estimates of alignment and quantification for a single RNA-seq sample with a sample size of 5.4 Gb also and runtime estimates for eQTL analyses and other steps in the workflow with genotype data for a single bovine chromosome (*Bos taurus* autosome 2) and expression counts from 88 samples. These runtime estimates provided valuable information to plan future analyses with a higher sample size based on the available computational infrastructure.

To our knowledge the eQTL-Detect is one of the initial attempts to develop a bioinformatics pipeline using workflow manager technology like Nextflow. Except for the eQTL-Catalogue pipeline (30) developed by human GTEx consortia (20), no other Nextflow or other workflow manger based eQTL detection pipelines are available in public domain like GitHub or other developer platforms for free download. Some non-workflow manager based solutions include SPIRE (31), eQTLQC (32) and eQTL detector (https://openaccess.uoc.edu/bitstream/10609/121606/6/martindiTFM0620memoria.pdf, last accessed in July 2024). Unlike our pipeline which supports fastq, bam and count matrices as input for expression data SPIRE only supports fastq file as input for expression data and eQTL detector only supports bam format. Similar to our pipeline eQTLQC also supports multiple format expression as input. However, it is not a modular based solution, which make it complex in terms of installation and usability for distribution of individual tasks of the pipeline for collaborative projects. Moreover, this tool only performs quality control of the input data and users have to perform eQTL detection with MatrixEQTL (33), which is not included with in the eQTLQC pipeline. Furthermore, by taking advantage of the eQTL-Detect modular design, it is possible to include some specialised tools like QTLIMP (34), eQTLMAPT (35) and eQTLMotiff (36) that perform specific post-eQTL studies with eQTL summary statistics. When the experiments were performed with limited sample size, QTLIMP can impute the missing eQTL associations. Focusing on *trans*-eQTL association eQTLMAPT performs post-eQTL multiple testing corrections with different permutation procedures. eQTLMotiff identifies the regulatory patterns across eQTLs by constructing a novel eQTL regulatory network by performing motif mining.

The eQTL-catalogue also developed using Nextflow and has many features similar to our pipeline. But the detection trans-eQTL was not implemented in eQTL-Catalogue. Unlike eQTL-Catalogue which implemented two separate pipelines for RNAseq data processing and QTL mapping, our pipeline can run all the analyses from input data processing (which include both RNAseq and genotype data processing) to eQTL detection with a single nextflow command (nextflow run main.nf -c conf/env_local.config -profile docker). This makes our pipeline more versatile and user-friendly compared to the available solutions for eQTL detection. We conducted a comparative analysis of eQTL-Detect and eQTL-Catalogue with the demo data provided in the BovReg/BovReg_eQTL GitHub page (https://github.com/BovReg/BovReg_eQTL) and using the same computational resources. As shown in the Table 2 and Supplemental Figure S1, no significant differences were found between the runtime estimates when each individual tasks of the pipeline are compared. Overall, the total run-time of eQTL-Detect is higher than the eQTL-Catalogue. This can be majorly attributed to the *trans*-eQTL analysis which is exclusive to eQTL-Detect and the minor difference between each individual tasks can be attributed to the type of tools used by these pipelines and the default parameters declared for each tool.

**Table 2.** The median run-time estimates (execution time in minutes) of different tasks in the two nextflow based pipelines eQTL-Detect and eQTL-Catalogue

Pipeline tasks	eQTL-Detect	eQTL-Catalogue
Reference Genome Indexing	29.3	25.9
Read trimming	0.1	1
Read alignment	2.9	0.4
Bam files sorting and merging	0.1	0.1
Quantification of gene expression	0.7	0.6
Merge expression counts	0.1	0.1
Extract sample genotypes	1.6	1.4
*cis*-eQTL nominal	0.2	0.01
*cis*-eQTL permutation	4	0.1
*trans*-eQTL nominal	7	NA
*trans*-eQTL permutation	7.1	NA
Total	53.1	29.61

The nextflow based scripts can be resumed from the point they stopped using the ‘-resume’ parameter (nextflow run main.nf -resume), if there is any interruption due to a server breakdown or other minor issues or if the user wants to edit some intermediate processes e.g. changing mapping parameters or correcting strandedness information of RNA-seq data sets. This feature is especially helpful when running module 1, for which runtime increases linearly from a few hours to months based on the number of input RNA-seq samples, and it saves lot of computational resources and time in the event of interruption. For collaborative projects it is possible to use the modular approach to run the entire pipeline independently by different partners and perform meta-analysis for eQTL detection by combining intermediate results using the last module (eQTL detection). This is advantageous, if the raw sequencing data could not be shared due to confidentiality issues or if project partners want to distribute the computational burden specifically associated with aligning the RNAseq samples. The major issue for sharable bioinformatics pipeline is to make it compatible across different computational infrastructures. We provided a list of most common nextflow config files (https://github.com/BovReg/BovReg_eQTL/tree/main/conf) to run the pipeline on different computer cluster environments along with the option to choose different container technologies.

This pipeline can be adopted to detect eQTLs in species other than *Bos taurus* by just changing the reference genome and annotation in the nextflow.config file. Based on the runtime estimates from our pilot run with 88 samples, to analyse a large number of samples it is advisable to run at least alignment (module_1_eQTLDetect.nf) on a grid or cloud based parallel computational solution. This is efficient as it significantly reduces the runtime, and the analyses of other modules can be deployed on a local server. Similar to the web based postGWAS (37) server, with the future eQTL-Detect updates, there is scope to include to include other association studies like aseQTL (allele specific expression QTL), genome-wide association studies (GWAS) and transcriptome-wide association studies (TWAS) as separate nextflow modules and also to include a wide variety of molecular phenotypes to make eQTL-Detect more comprehensive and complete go to pipeline for all kind of association studies.

## Supplementary Material

lqae122_Supplemental_File

## Data Availability

Sequencing data are available under PRJEB34570 and PRJEB33849. The eQTL-Detect pipeline scripts and demonstration data can be downloaded from BovReg GitHub page (https://github.com/BovReg/BovReg_eQTL) and Zenodo (https://zenodo.org/doi/10.5281/zenodo.8305279).

## Notes

Present address: Praveen Krishna Chitneedi, Research Institute for Farm Animal Biology (FBN), Wilhelm-Stahl-Allee 2, Dummerstorf, Mecklenburg–Western Pomerania 18196, Germany.
