# Supplementary material for: eQTL-Detect: nextflow-based pipeline for eQTL detection in modular format with sharable and parallelizable scripts
Source: NAR Genom Bioinform. 2024 Sep 24;6(3):lqae122. doi: 10.1093/nargab/lqae122 (PMC11420669; doi:10.1093/nargab/lqae122)
Supplement: lqae122_Supplemental_File [file lqae122_supplemental_file.pdf]

Figure S1. Run-time estimates of different tasks from the pipelines eQTL-Catalogue and eQTL-Detect when running with the same demo-data. The sub-plots a) and b) shows the run-time estimates of different tasks from eQTL-Catalogue pipeline and the sub-plots c) , d) and e) shows the run-time estimates of different tasks from eQTL-Detect pipeline.

a)

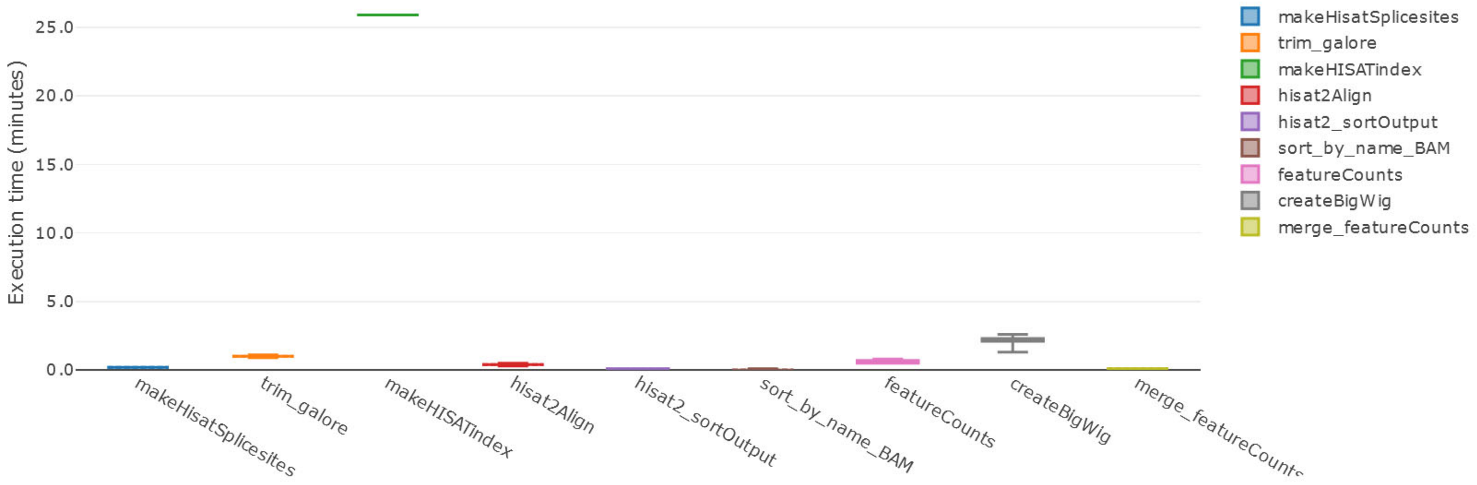

b)

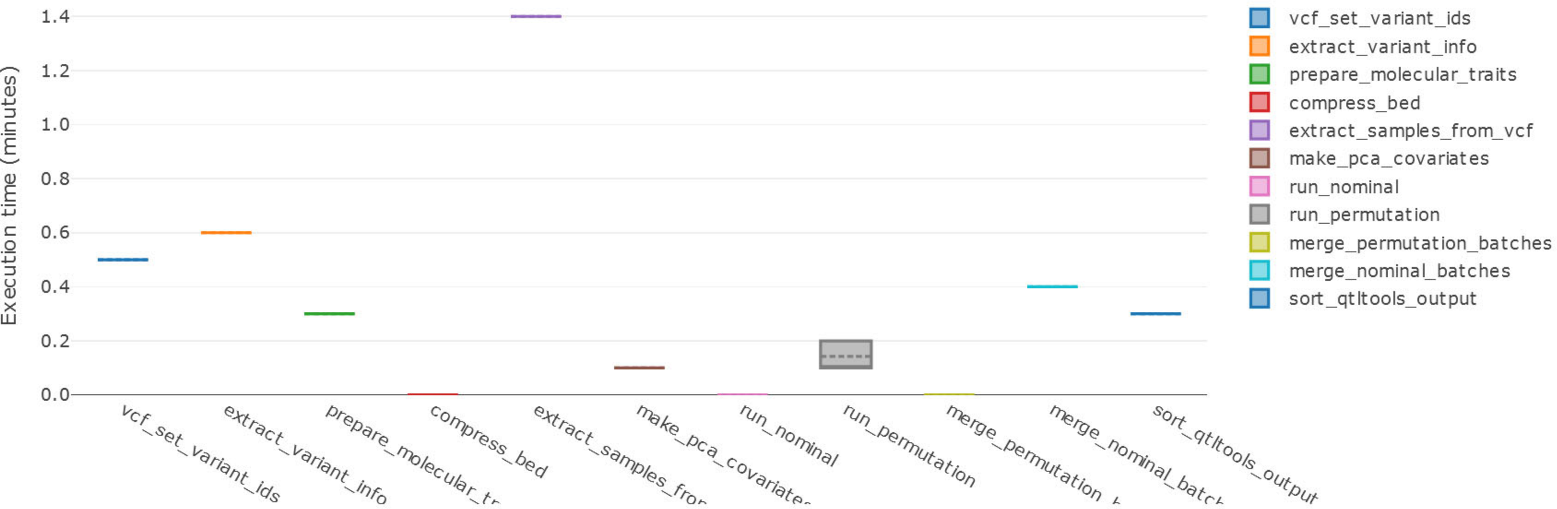

c)

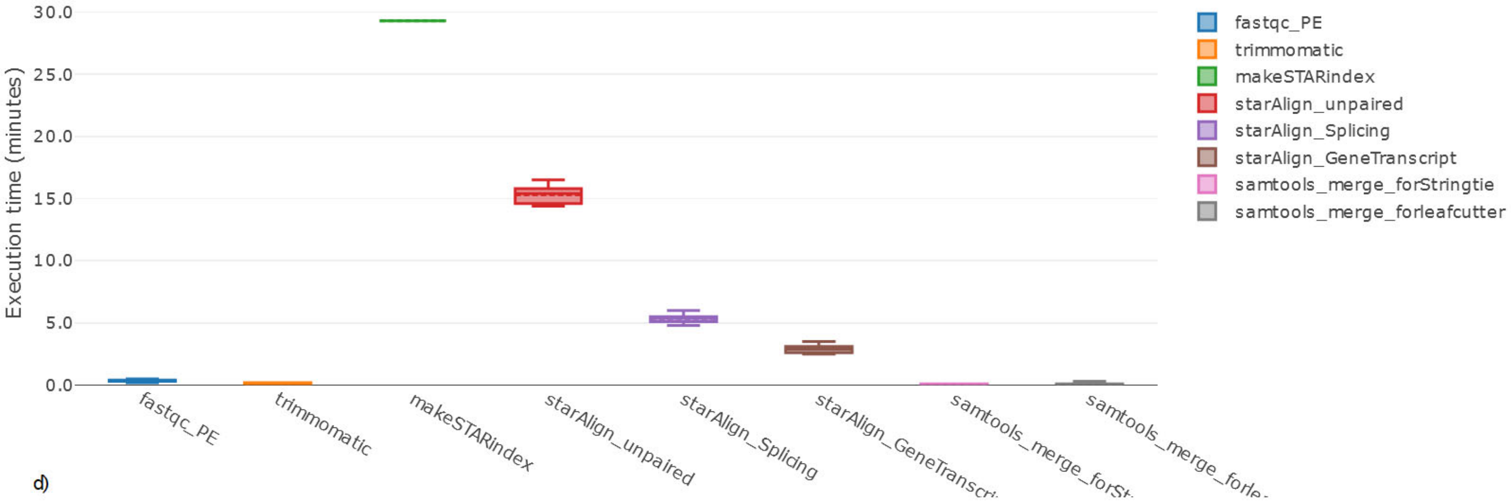

Execution time (minutes)
fastqc_PE
trimmomatic
makeSTARindex
starAlign_unpaired
starAlign_Splicing
starAlign_GeneTranscript
samtools_merge_forStringtie
samtools_merge_forleafcutter

d)

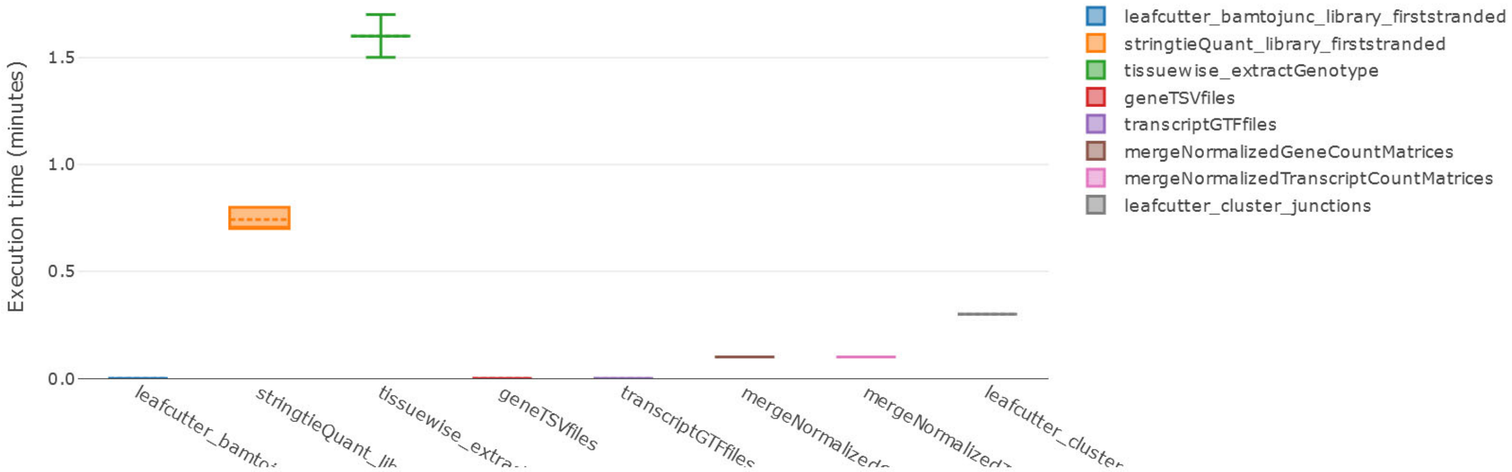

Execution time (minutes)
leafcutter_bamtojunc_library_firststranded
stringtieQuant_library_firststranded
tissuewise_extractGenotype
geneTSVfiles
transcriptGTFfiles
mergeNormalizedGeneCountMatrices
mergeNormalizedTranscriptCountMatrices
leafcutter_cluster_junctions

e)

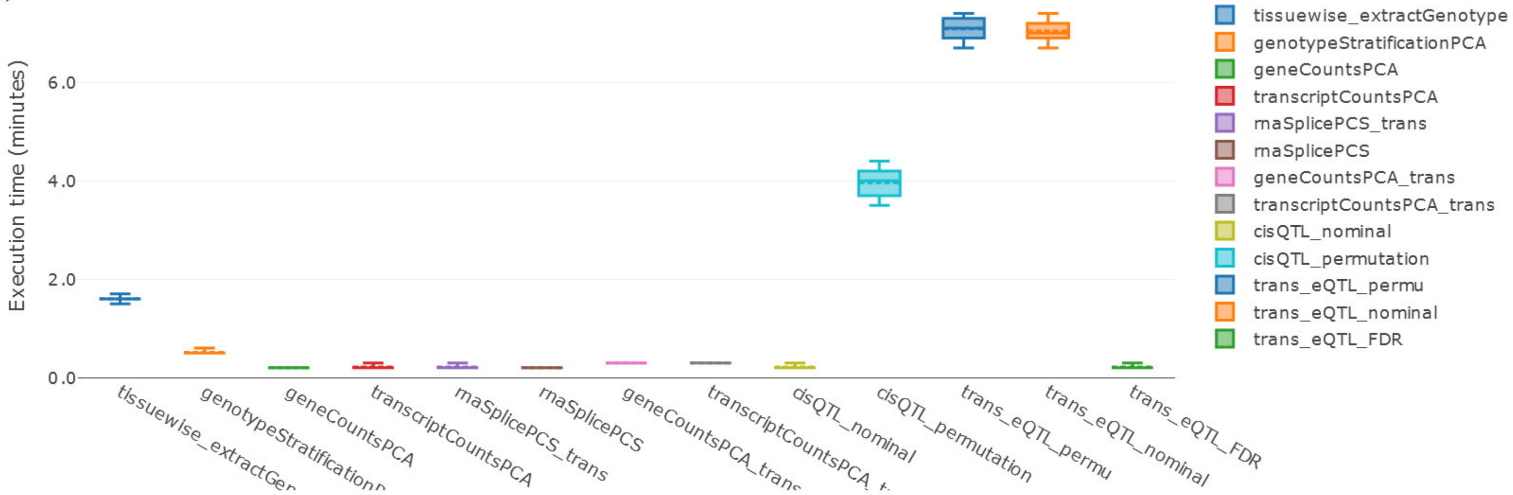

Execution time (minutes)
tissuewise_extractGenotype
genotypeStratificationPCA
geneCountsPCA
transcriptCountsPCA
rnaSplicePCS_trans
rnaSplicePCS
geneCountsPCA_trans
transcriptCountsPCA_trans
cisQTL_nominal
cisQTL_permutation
trans_eQTL_permu
trans_eQTL_nominal
trans_eQTL_FDR
